# Highly Efficient Targeted Gene Editing in Upland Cotton Using the CRISPR/Cas9 System

**DOI:** 10.3390/ijms19103000

**Published:** 2018-10-01

**Authors:** Shouhong Zhu, Xiuli Yu, Yanjun Li, Yuqiang Sun, Qianhao Zhu, Jie Sun

**Affiliations:** 1 The Key Laboratory of Oasis Eco-Agriculture, College of Agriculture, Shihezi University, Shihezi 832000, Xinjiang, China; zhushouhong2014@126.com (S.Z.); axiu985211@163.com (X.Y.); lyj20022002@sina.com.cn (Y.L.); 2 Key Laboratory of Plant Secondary Metabolism and Regulation of Zhejiang Province, College of Life Sciences, Zhejiang Sci-Tech University, Hangzhou 310016, Zhejiang, China; sunyuqiang@zstu.edu.cn; 3 CSIRO Agriculture and Food, GPO Box 1700, Canberra 2601, Australia

**Keywords:** CRISPR/Cas9, genome editing, cotton, *GhALARP*, mutation

## Abstract

The clustered regularly interspaced short palindromic repeats/CRISPR-associated protein 9 (CRISPR/Cas9) gene editing system has been shown to be able to induce highly efficient mutagenesis in the targeted DNA of many plants, including cotton, and has become an important tool for investigation of gene function and crop improvement. Here, we developed a simple and easy to operate CRISPR/Cas9 system and demonstrated its high editing efficiency in cotton by targeting-ALARP, a gene encoding alanine-rich protein that is preferentially expressed in cotton fibers. Based on sequence analysis of the target site in the 10 transgenic cottons containing CRISPR/Cas9, we found that the mutation frequencies of *GhALARP-A* and *GhALARP-D* target sites were 71.4–100% and 92.9–100%, respectively. The most common editing event was deletion, but deletion together with large insertion was also observed. Mosaic mutation editing events were detected in most transgenic plants. No off-target mutation event was detected in any the 15 predicted sites analyzed. This study provided mutants for further study of the function of *GhALARP* in cotton fiber development. Our results further demonstrated the feasibility of use of CRISPR/Cas9 as a targeted mutagenesis tool in cotton, and provided an efficient tool for targeted mutagenesis and functional genomics in cotton.

## 1. Introduction

Increasing yield and quality of crops is the ultimate objective of plant breeding. Conventional plant breeding has made a significant contribution to meet the increasing demands on crop products caused by rapid expansion of human population, but it will be more difficult to keep up with the increasing demands of humans and industries for the agricultural products while under the pressure of the global environmental challenges that we face. Modern biotechnology and molecular approaches have been applied to plant breeding to promote plant genome manipulation and enhance selection of desired agronomic traits and performance of crops. However, the revolutionized molecular tools that are expected to change the way we breed new crops are the gene editing technologies [1,2], including zinc finger nucleases (ZFNs) [3,4,5], transcription activator-like effect nucleases (TALENs) [6,7,8,9], and clustered regularly interspaced short palindromic repeats/CRISPR-associated protein (CRISPR/Cas) [10,11], particularly the CRISPR/Cas9 system. Compared with ZFNs and TALENs, the CRISPR/Cas9 system has many advantages, such as being easy to design and operate, having higher targeted editing efficiency, being cheaper, and having the ability to complete multiplex genome editing [12,13,14]. It has become the most powerful and popular tool for functional genomics and targeted mutagenesis to create mutations that can be directly used in plant breeding.

CRISPR/Cas9 mainly utilizes single guide RNA (sgRNA) to direct cleavage of the target DNA by the CAS9 protein, which generates double-stranded breaks (DSBs) at the target site that are usually repaired through nonhomologous end joining (NHEJ), an error-prone mechanism causing mutations in the target site [15,16,17]. This technology has been applied in *Arabidopsis* [18,19], rice [20,21], wheat [12], corn [22,23], tobacco [10,24], tomato [25], Sorghum bicolor [26], and other monocotyledonous and dicotyledonous plants [27,28,29,30] to generate mutants for investigation of gene function and new germplasms for crop breeding. Several studies have also investigated the feasibilities of the use of CRISPR/Cas9 in cotton (*Gossypium hirsutum*), an allotetraploid species with a large genome (~2.5 Gb) [31,32]. These studies demonstrated a moderate to very high editing efficiency of CRISPR/Cas9 for both exogenous marker genes [33,34] and endogenous genes in cotton [34,35,36,37,38]. Successful use of CRISPR/Cas9 in cotton still relies on *Agrobacterium*-mediated transformation and tissue culture, a genotype-dependent and low efficiency process, but it provides a powerful tool for cotton functional genomics as CRISPR/Cas9 seems to be more efficient than RNA interference (RNAi) and virus induced gene silencing (VIGS) in terms of knocking out the function of target genes [36]. CRISPR/Cas9 has been used to edit a couple of agronomically important cotton genes, such as *MYB25-like* [37] and a gene encoding arginase (*ARG*) [38], but it is necessary to further develop an efficient gene editing system for molecular biology studies that can be easily adopted by any laboratory with basic facilities and to use more cotton genes with potential breeding value in gene editing studies.

In this study, we used a modified, simple, and easy to operate CRISPR/Cas9 system to generate targeted mutations in *GhALARP*, a gene preferentially expressed in cotton fibers that encodes a protein that is rich in alanine. Our major aim is to investigate the feasibility of a simple CRISPR/Cas9 gene editing system in targeted mutagenesis in cotton, and in future, to have transgenic cottons with different types of mutations in a gene with a potential role in fiber development for functional characterization.

## 2. Result

### 2.1. Construction of the CRISPR/Cas9-GhALARP-sgRNA Vector

**Figure 1:**
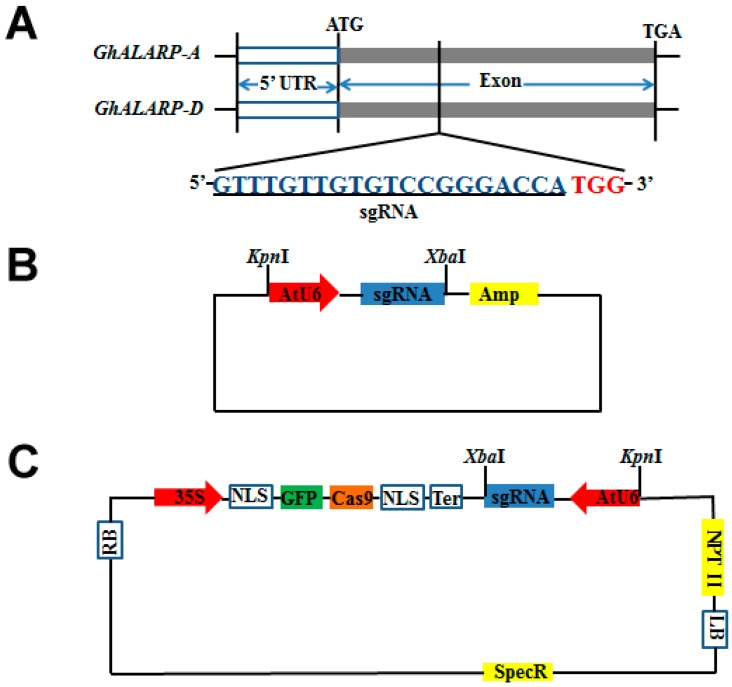
Schematic diagram of the 35S:Cas9/single guide RNA (sgRNA) vector. (**A**) Gene structures of *GhALARP-A* and *GhALARP-D*, and the sgRNA sequence (blue nucleotides) targeting both *GhALARP-A* and *GhALARP-D*. White boxes indicate 5′-untranslated region (UTR) and the promoter region; gray boxes indicate exons; red nucleotides indicate the protospacer adjacent motif (PAM) sequence; (**B**) Composition of the *AtU6*-sgRNA vector. *AtU6*, promoter of the *U6* gene of *Arabidopsis thaliana*; (**C**) Genetic map of the 35S:Cas9/sgRNA construct. Two nuclear localization sequences (NLSs) at the N terminal and C terminal of the green fluorescent protein (GFP)-Cas9 were originated from SV40 NLS-nuclear localization signal (SV40 large T antigen) and nucleoplasmin NLS-bipartite nuclear localization signal (nucleoplasmin), respectively. LB: left border; RB: right border.

One objective of this study was to have a simple protocol for molecular biology studies that could be adopted by any laboratory with basic facilities. To this end, we developed a two-step protocol that can generate in four days a CRISPR/Cas9-sgRNA construct by standard restriction enzyme digestion and ligation. The first step was to assemble an intermediate *AtU6*:sgRNA vector by recombining a double-stranded sgRNA with a modified commercial vector (based on the pEASY-Blunt-Zero vector) that contains the *AtU6* promoter. This was achieved by digestion of the modified commercial vector with *Bbs*I and ligation of it with the double-stranded sgRNA containing adaptor sequences compatible with *Bbs*I (Figure 1A,B). The use of an sgRNA intermediate expression vector facilitated the assembly process to ensure the expression of the sgRNA. In the next step, the *AtU6*:sgRNA cassette was recombined into the 35S:Cas9 expression vector by digesting the *AtU6*:sgRNA intermediate expression vector and the 35S:Cas9 vector with *Kpn*I and *Xba*I, and ligating the *AtU6*:sgRNA fragment (~230 bp) with the linearized 35S:Cas9 vector (Figure 1C). Compared to the previous approach used in cotton [34], this construction procedure is easy to operate, simple, and inexpensive.

In this study, we used *GhALARP* as a target gene to evaluate the construction protocol and the editing efficiency of the 35S:Cas9-sgRNA construct. *GhALARP* is predominantly expressed in cotton fibers and is isolated based on a messenger RNA (mRNA) fluorescence differential display. It is a 201 bp long single exon gene, and encodes a protein enriched in alanine. The two homoeologs of *GhALARP*, *Gh_A09G1166,* and *Gh_D09G1172* [32], were named as *GhALARP-A* and *GhALARP-D*, respectively. They have identical coding sequences (Figure 1A; Supplementary Figure S1). To simultaneously edit both *GhALARP-A* and *GhALARP-D*, a single sgRNA targeting the coding sequence of *GhALARP* was designed (Figure 1A and Supplementary Figure S1).

### 2.2. Analysis of CRISPR/Cas9-sgRNA-Mediated Mutagenesis in GhALARP-A and GhALARP-D

**Figure 2:**
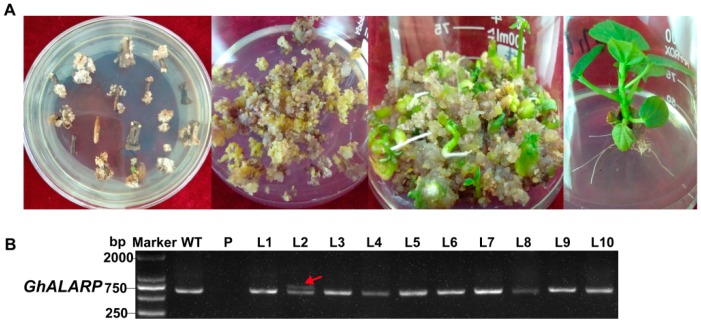
Cotton transformation and analysis of the target site. (**A**) The regeneration process of cotton transgenic plants; (**B**) Gel analysis of the PCR products amplified from the target sites of the 10 independent transgenic cotton plant events (L1 to L10). Lanes wild type (WT) (648 bp) and P are the PCR products from the non-transformed wild-type plant YZ-1 (negative control for the target site) and the 35S:Cas9 construct (positive control for the target site), respectively. The red arrow shows the PCR product with a potentially relatively-large insert in the target site.

To generate CRISPR/Cas9-sgRNA-mediated gene editing events, we infected hypocotyls from young cotton seedlings (cv. YZ-1) with *Agrobacterium tumefaciens* (strain LB4404) containing the CRISPR/Cas9-sgRNA editing system. Embryogenic calli were generated after several subcultures in the presence of antibiotic *Kanamycin*. The embryonic calli produced a large number of cotyledonary embryos after propagation and differentiation, and eventually differentiated into seedlings (Figure 2A). To investigate the editing events in the regenerated cotton plantlets, ten transgenic lines (L1 to L10) with the transfer DNA (T-DNA) from 12 independent regenerated plants were used in further analysis of their target sites and flanking sequences.

As the coding region of *GhALARP-A* and *GhALARP-D* are identical, to distinguish the two homoeologs of *GhALARP*, we designed one of the primers in the promoter region of *GhALARP* so that the two homoeologs can be separated based on the single nucleotide polymorphisms (SNPs) located in their promoters. In total there were 13 SNPs (Supplementary Figure S1).

**Figure 3:**
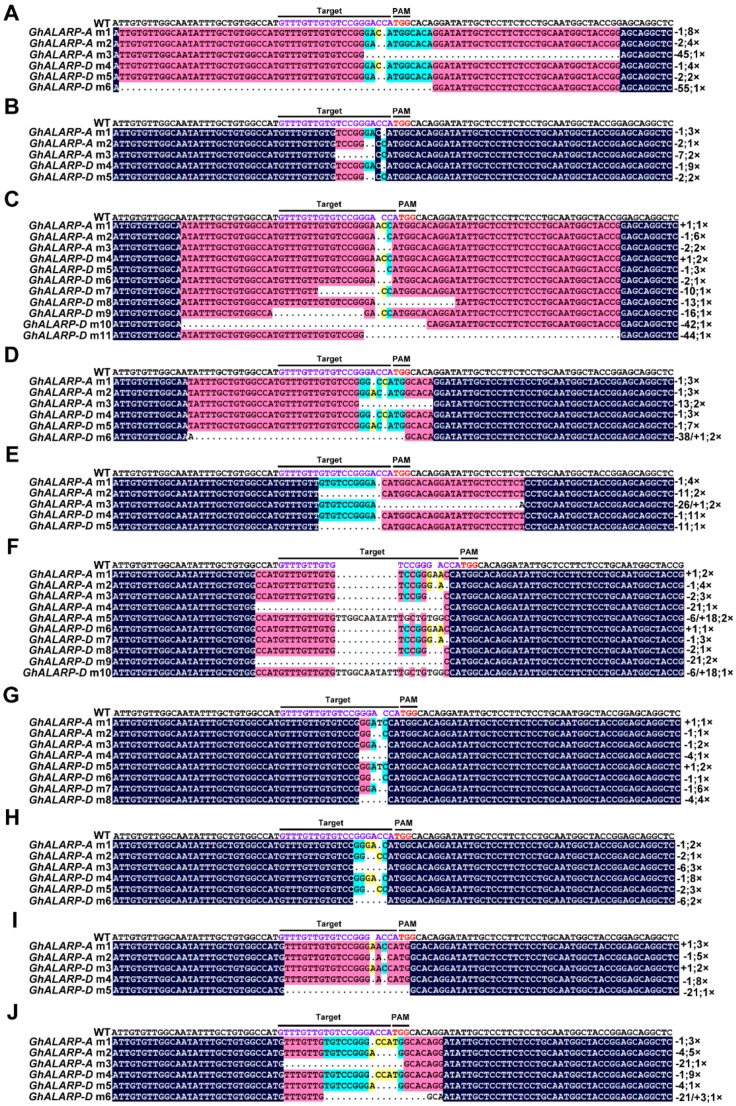
Sequence analysis of the target sites in *GhALARP-A* and *GhALARP-D*. (**A**–**J**) Alignment of genomic sequences of the target site and its flanking regions from the transgenic cotton lines (L1 to L10). The purple nucleotides indicate the target sequences of sgRNA in *GhALARP*. The red nucleotides indicate the PAM sequence. The frequency of each mutation and the mutation types are shown on the right.

We obtained a specific PCR product from each of the 10 transgenic lines with a similar size to that amplified in the wild-type; however, a slightly bigger size product was also amplified in plant L2 (Figure 2B), suggesting a possible large insert in the target site in this plant. To investigate the details of sequence changes in the target site, for each transgenic plant, we sequenced 20 randomly selected colonies and found mutations in the target sites of both *GhALARP-A* and *GhALARP-D* in each plant (Figure 3 and Supplementary Figure S2, Table 1). Of the 200 sequences analyzed, only four sequences (two and one *GhALARP-A* sites of L7 and L9, respectively, and one *GhALARP-D* site of L8) were unchanged. The mutation frequencies were 71.4–100% and 92.9–100% for the target sites of *GhALARP-A* and *GhALARP-D*, respectively (Table 1).

The mutated events were grouped into three types based on the characteristics of sequence changes: (1) a single nucleotide insertion (+1 bp), (2) one or multiple nucleotide deletions (−1 bp, −2 bp, −4 bp, −6 bp, −7 bp, −10 bp, −11 bp, −13 bp, −16 bp, −21 bp, −42 bp, −44 bp, −45 bp, and −55 bp), and (3) a mixture of nucleotide deletion and insertion (−21/+3 bp, −26/+1 bp, −6/+18 bp, −38/+1 bp, and −13/+99 bp) (Table 1). Except plants L1 and L8, for which only a type 2 editing event was observed, at least two different editing types were found in the other eight plants (Figure 3, Supplementary Figure S2). For example, plant L6 contained all three types. Nucleotide deletion seemed to be the major type of gene editing event. The longest deletion was 55 bp observed in *GhALARP-D* of plant L1. Sequencing confirmed a large insertion in the longer PCR product observed in plant L2, which was a result of a 13 bp deletion and 99 bp insertion (Figure 2B, Supplementary Figure S2). These results suggest that, in addition to small indels, the CRISPR/Cas9-sgRNA system is able to induce complicated sequence changes in the target site.

In addition to the similar editing efficiency observed in *GhALARP-A* and *GhALARP-D*, it seems that the CRISPR/Cas9-sgRNA system also induced similar mutations in *GhALARP-A* and *GhALARP-D*, as all plants except for L3 had a similar number of different mutations in the two homoeologs (Figure 3). In all 10 plants analyzed, two or more different mutations were observed for both *GhALARP-A* and *GhALARP-D*. For plants L7 and L9, the four and two different mutations observed in *GhALARP-A* were likely to be mosaic mutations as unchanged wild-type *GhALARP-A* target sequences were also detected. Similarly, for plant L8, its three mutations observed in *GhALARP-D* were also likely to be mosaic mutations. For plant L5, its two mutations observed in *GhALARP-D* were likely to be bi-allelic ones because no unchanged *GhALARP-D* target site was detected. For all others, the observed multiple mutations could be a combination of mosaic and bi-allelic mutations.

### 2.3. Off-Target Analysis in the Transgenic Cotton Lines

**Figure 4:**
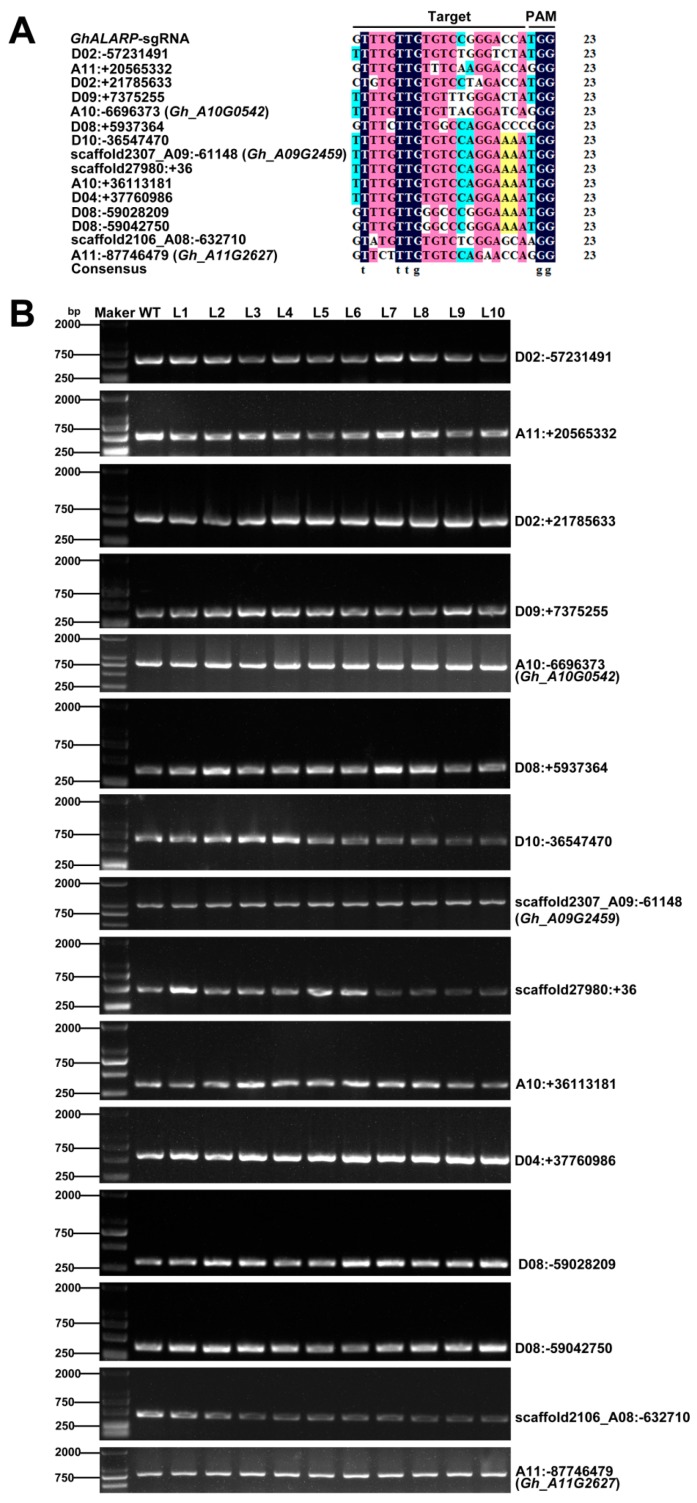
Analysis of the 15 potential off-target sites in the transgenic lines with verified editing in *GhALARP***.** (**A**) Alignment of the 15 putative off-target sites and the *GhALARP*-sgRNA sequence; (**B**) PCR amplification results of the DNA sequences containing the predicted off-target. From the top to the bottom panels, D02: −57231491 (572 bp), A11: +20565332 (482 bp), D02: +21785633 (574 bp), D09: +7375255 (352 bp), A10: −6696373 (*Gh_A10G0542*) (748 bp), D08: +5937364 (364 bp), D10: −36547470 (664 bp), scaffold2307_A09: −61148 (*Gh_A09G2459*) (940 bp), scaffold27980: +36 (476 bp), A10: +36113181 (340 bp), D04: +37760986 (563 bp), D08: −59028209 (317 bp), D08: −59042750 (367 bp), scaffold2106_A08: −632710 (445 bp), and A11: −87746479 (*Gh_A11G2627*) (759 bp). Lanes 1–10 represent ten independent transgenic cotton plants (L1 to L10). Lanes WT is the PCR product from the non-transformed wild-type plant YZ-1 (negative control for the target site).

The occurrence of off-target mutations is a major concern affecting the use of the gene editing technology in plant functional genomics and molecular breeding. To assess the possible off-target effects of CRISPR/Cas9-*GhALARP*-sgRNA, we first predicted potential off-targets of *GhALARP*-sgRNA in the *G. hirsutum* genome [39]. A total of 19 potential off-target sites was computationally predicted (Supplementary Table S1). We then analyzed the sequences generated from 15 of the 19 potential off-target sites (four potential off-target sites (scaffold222153: −146, scaffold187744: −240, scaffold208662: +246, and D08: −59033434) were not analyzed because sequences around the sites were not good for primer design or were a potential artifact of genome assembly) in the 10 transgenic cotton plants containing Cas9, and we found no mutation in each site (Figure 4 and Supplementary Figure S3, Table 2). This result indicated that CRISPR/Cas9-sgRNA is highly specific in terms of target selection in cotton.

## 3. Discussion

Compared with ZFNs and TALENs, the CRISPR/Cas9 gene editing system is simple in design and operation, and it has a higher efficiency of target site editing. It has been widely used in basic and applied research in many plant species [40,41,42]. With the publication of the cotton genome sequences (*G. hirsutum*) [31,32], study of cotton has entered the post-genome era, in which one of the main objectives is to characterize and understand the functions of cotton genes. Rapid progress of the CRISPR/Cas9 gene editing technology provides an efficient tool for achieving this goal [10,11]. Several reports have been published on the use of CRISPR/Cas9 in targeted mutagenesis in cotton [33,34,35,36,37,38]. These studies demonstrated the efficiency of CRISPR/Cas9-mediated gene editing in cotton; however, developing new CRISPR/Cas9 gene editing systems and use of them in targeting more cotton genes are still the two key aspects that should be explored. In this study, we developed an easy to operate and highly effective CRISPR/Cas9 gene editing system for cotton functional genomics.

The design of sgRNA is one of the keys determining the success of CRISPR/Cas9 [43,44]. To monitor Cas9:sgRNA induced mutations, one way is to use sgRNA with a restriction site at the expected cleavage site so that the mutated allele can be distinguished from the un-mutated allele by digestion of the PCR products containing the target site [23,35,36]; another option is to sequence the PCR products to directly compare sequence changes [37]. The latter is not usually favored as a new editing construct due to its yet-to-be-confirmed editing ability, but we found it worked very well in our case. Of the 200 clones analyzed, only four were found to be un-edited (Table 1), suggesting that for carefully selected sgRNA, it is not necessary to have a restriction enzyme site. In doing so, it actually allows more choice for selection of optimized sgRNAs to achieve a high mutation rate in the target site [23], as demonstrated by this study.

*Agrobacterium*-mediated genetic transformation is the most widely used method in cotton, although only a few cotton genotypes are transformable [45]. To ensure success of gene editing in the stable transgenic cotton plants, the CRISPR/Cas9-sgRNA system was, in some cases, tested and verified in protoplasts [35] or using a transient expression approach [36]. It was found that nucleotide substitution and deletion were the most common mutations observed in protoplasts and stable transgenics, respectively; the nature of mutations thus seemed to be different in these two approaches [35]. The editing efficiency of *Agrobacterium*-mediated genetic transformation was also higher in cotton hypocotyls than in protoplasts [35]. This is likely related to the longer time period of the transformation process. In this study, we used the hypocotyl to generate transgenics containing CRISPR/Cas9-sgRNA, and detected mutations in all the 10 independent positive transgenic plants (Figure 2B and Figure 3). We classified the mutations into three types: a single nucleotide insertion, a single or multiple nucleotide deletion, and a mixture of nucleotide deletion/insertion (Table 1). Consistent with the results observed in other plants as well as in cotton, nucleotide deletion was the main type of mutation [10,22,33,34,35,36,37,38,46,47,48]; nevertheless, we also observed a large insert (99 bp) in one of the 10 transgenic plants (Supplementary Figure S2), which has not been previously reported in cotton. We found that the 99 bp insert and its upstream five nucleotides (GTTGT, part of the target site) is fully matched with a sequence fragment on chromosome D09, on which *GhALARP-D* resides, suggesting that the 5 bp micro-homologous sequence might play a role in generation of the large insertion, although the exact mechanism responsible for this outcome is yet to be uncovered.

Editing efficiency and off-target effect are the two key considerations when using the CRISPR/Cas9 gene editing tool [20,30,40]. In this study, all 10 independent transgenic plants containing CRISPR/Cas9 were found to be edited in the target site, indicating that we have achieved an editing efficiency similar to or higher than that previously reported in cotton [34,35,36,37]. Variable editing efficiency reported in different studies may be related to a variety of factors, such as use of different promoter-driven sgRNAs [23,34], the specificity of the designed sgRNA [43,44], and the transformation methods used [35,36]. Similar to other studies in cotton [34,35,36,37], no off-target event was detected in all 15 potential off-target sites (Figure 4 and Supplementary Figure S3, Table 2).

This study used *GhALARP*, a gene predominately expressed in cotton fibers, as a target of gene editing. A single sgRNA was designed to simultaneously target the identical sequence of *GhALARP*-*A* and *GhALARP*-*D* (Figure 1 and Supplementary Figure S1). As expected, both homoeologs were found to be mutated with a similar mutation rate and types of mutations (Figure 3). However, when it is necessary to investigate the sub-functionalities of the At and Dt subgenome homoeologs, it requires specific mutation in only one of the homoeologs. As an allotetraploid species, the two homoeologs of each cotton gene are usually highly similar, making it hard to select specific target sequences for RNAi and VIGS [49,50], the two main approaches for investigation of gene function in cotton, as both approaches require a relatively large sequence fragment as a target and most cotton homoeologous genes might not be different enough to have such a specific target. In contrast, gene editing only needs a 20 bp sequence as a target and is sensitive to mismatches between the sgRNA and its target, therefore, specific targeting of one of the two homoeologs can usually be relatively easily achieved. In addition, the study has also shown that CRISPR/Cas9 is able to generate mutants having more obvious and unified phenotypes than VIGS [36], indicating that gene editing would be superior to RNAi for cotton functional genomics.

In conclusion, this study developed an easy to operate and highly efficient CRISPR/Cas9 system and provided an efficient tool for targeted mutagenesis and functional genomics in cotton.

## 4. Materials and Methods

### 4.1. Plant Material and Growth

Upland cotton (*G. hirsutum* L.) cultivar of ‘YZ-1’ was used in this study. The regenerated cotton plantlets were grown in the nutrient soil mixed with vermiculite (the ratio of nutrient soil and vermiculite was 3:1 (*V*/*V*), the pot size (the upper diameter (cm) * the lower diameter (cm) * the height (cm) = 23*18*21.5)), and the growth chambers with a light regime of 16 h light/8 h dark at 28 °C in Shihezi University (Shihezi, China).

### 4.2. Selection of sgRNA Targeting GhALARP

The *GhALARP* gene preferentially expressed in cotton fibers and encoding a protein that is rich in alanine was selected as a target of gene editing. Its corresponding copies in the At and Dt subgenomes of *G. hirsutum* are *Gh_A09G1166* (*GhALARP-A*) and *Gh_D09G1172* (*GhALARP-D*) [32] from the CottonGen website (available online: https://www.cottongen.org), respectively.

Selection of sgRNA targeting *GhALARP* was performed based on the approach and rules previously described [39,48]. *GhALARP* is a single exon gene. Both homoeologs of *GhALARP* have identical sequences; the sgRNA selected was thus expected to target both *GhALARP-A* and *GhALARP-D* (Figure 1A).

### 4.3. Construction of 35S-Cas9-AtU6-GhALARP-sgRNA Vector

The expression vector was constructed in two steps. The first step involved creation of the *AtU6*-sgRNA vector by ligation of the double stranded sgRNA with a linearized intermediate expression vector (cut by *Bbs*I, New England BioLabs, Ipswich, MA, USA) containing the promoter of *Arabidopsis thaliana U6* (*AtU6*). The intermediate expression vector was modified from the pEASY-Blunt-Zero vector (TransGen, Beijing, China) by adding *AtU6* followed by restriction recognition sites of *Bbs*I and *Xba*I (Figure 1B). The double stranded sgRNA used in ligation was prepared by slowly annealing together the following two complementary oligoes: sgRNA-F, 5′-GATTGTTTGTTGTGTCCGGGACCA-3′ and sgRNA-R, 5′-AAACTGGTCCCGGACACAACAAAC-3′ (the underlined nucleotides are the adaptor sequences complementary to the sticky ends of the intermediate expression vector after *Bbs*I digestion) using the following temperature steps in a PCR machine: 95 °C 2 min, 70 °C 10 min, 55 °C 10 min, 40 °C 10 min, and 25 °C 10 min.

The second step was to move the *AtU6*-sgRNA fragment from the *AtU6*-sgRNA vector to the CRISPR/Cas9 vector. To do that, both vectors were digested completely with *Kpn*I and *Xba*I. The purified *AtU6*-sgRNA fragment was then ligated with the linearized CRISPR/Cas9 vector to construct the final 35S-Cas9-AtU6-sgRNA gene editing vector (Figure 1C).

### 4.4. Agrobacterium-Mediated Transformation of Cotton

*Agrobacterium* (strain LB4404) harboring the 35S-Cas9-AtU6-*GhALARP*-sgRNA vector was used to infect cotton hypocotyls. Calli induction and plant regeneration were performed according to the published protocol [51].

### 4.5. Mutant Identification and Analysis of Genomic Target Site

Cotton genomic DNA was extracted by Plant Genomic DNA Kit (TIANGEN, Beijing, China) according to the manufacturer’s instructions. To detect mutations in the target site, PCR primers up-stream and down-stream of the target site in the *GhALARP* gene were designed, which amplified a genomic fragment of 648 bp containing the target site. The primer sequences were *GhALARP*-F: 5′-GTGGCCCACGTATCAAAGT-3′ and *GhALARP*-R: 5′-ATGCACCAAGAGAGCAATT-3′. The PCR reaction contained 1× PrimsSTAR buffer (Mg^2+^ Plus), 200 μM dNTP, 1.25 U PrimeSTAR HS DNA Polymerase (TaKaRa, Dalian, China), 100 ng DNA template, and 0.2 μM of each primer, and was subjected to a regime of 32 cycles of 98 °C for 10 s, 55 °C for 15 s, and 72 °C for 40 s. PCR products were purified and cloned into pEASY-Blunt cloning vector (Transgen, Beijing, China). For each transgenic plant, plasmid DNA was extracted from randomly selected 20 colonies and sequenced (BGI, Beijing, China). Sequences were analyzed using the DNAMAN software.

### 4.6. Analysis of Possible Off-Target Sites

All potential off-target sites were predicted using online software (http://cbi.hzau.edu.cn/cgi-bin/CRISPR) [39]. All were selected for primer design but only 15 of the 19 putative off-target sites were analyzed. The remaining four (scaffold187744: −240, scaffold208662: +246, D08: −59033434 and scaffold222153: −146) had poor sequences around the predicted target sites or were a potential artifact of genome assembly and were not analyzed. Each potential off-target site has 4 or 5 mismatches with the *GhALARP*-sgRNA (Figure 4A). To detect the potential off-target cleavage event, primers amplifying a genomic fragment containing the predicted off-target site were designed for each gene. The primer sequences are shown in Supplementary Table S2. The amplified PCR products were purified and cloned into pEASY-Blunt cloning vector (Transgen, Beijing, China). For each of the ten independent transgenic cotton plants, five or more randomly selected colonies were sequenced (BGI, Beijing, China) and analyzed as aforementioned.

## Figures and Tables

**Table 1:** Percentage of different mutation events in *GhALARP* induced by CRISPR/Cas9-sgRNA.

Sample	Gene	Rate of Different Nucleotide Insertions (+) and Deletions (−) (%)																				
		0	+1	−1	−2	−4	−6	−7	−10	−11	−13	−16	−21	−42	−44	−45	−55	−21/+3	−26/+1	−38/+1	−6/+18	−13/+99
L1	*GhALARP-A*			61.5	30.8											7.7						
	*GhALARP-D*			57.1	28.6												14.3					
L2	*GhALARP-A*			50	16.7			33.3														
	*GhALARP-D*			64.3	14.3																	21.4
L3	*GhALARP-A*		11.1	66.7	22.2																	
	*GhALARP-D*		18.2	27.3	9.1				9.1		9.1	9.1		9.1	9.1							
L4	*GhALARP-A*			75							25											
	*GhALARP-D*			83.3																16.7		
L5	*GhALARP-A*			50						25									25			
	*GhALARP-D*			91.7						8.3												
L6	*GhALARP-A*		16.7	33.3	25								8.3								16.7	
	*GhALARP-D*		12.5	37.5	12.5								25								12.5	
L7	*GhALARP-A*	28.6	14.3	42.9		14.3																
	*GhALARP-D*		15.4	53.8		30.8																
L8	*GhALARP-A*			33.3	16.7		50															
	*GhALARP-D*	7.1		57.1	21.4		14.3															
L9	*GhALARP-A*	11.1	33.3	55.6																		
	*GhALARP-D*		18.2	72.7									9.1									
L10	*GhALARP-A*			33.3		55.6							11.1									
	*GhALARP-D*			81.8		9.1												9.1				

**Table 2:** Mutation detection and analysis of the 15 potential off-target sites in the 10 independent transgenic cotton lines.

sgRNA Name	Putative Off-Target	No. of Mismatched Nucleotides	No. of Examined Events	No. of Off-Target Events
*GhALARP*-sgRNA	D02: −57231491	4	86	0
*GhALARP*-sgRNA	A11: +20565332	4	61	0
*GhALARP*-sgRNA	D02: +21785633	4	63	0
*GhALARP*-sgRNA	D09: +7375255	4	74	0
*GhALARP*-sgRNA	A10: −6696373 (*Gh_A10G0542*)	5	100	0
*GhALARP*-sgRNA	D08: +5937364	5	79	0
*GhALARP-sgRNA*	D10: −36547470	4	82	0
*GhALARP*-sgRNA	scaffold2307_A09: −61148 (*Gh_A09G2459*)	4	100	0
*GhALARP*-sgRNA	scaffold27980: +36	4	73	0
*GhALARP*-sgRNA	A10: +36113181	4	66	0
*GhALARP*-sgRNA	D04: +37760986	4	92	0
*GhALARP*-sgRNA	D08: −59028209	4	65	0
*GhALARP*-sgRNA	D08: −59042750	4	88	0
*GhALARP*-sgRNA	scaffold2106_A08: −632710	5	83	0
*GhALARP*-sgRNA	A11: −87746479 (*Gh_A11G2627*)	5	100	0

## Supplementary Materials

Supplementary materials can be found at http://www.mdpi.com/1422-0067/19/10/3000/s1.
