# Supplementary material for: Highly Efficient Targeted Gene Editing in Upland Cotton Using the CRISPR/Cas9 System
Source: Int J Mol Sci. 2018 Oct 1;19(10):3000. doi: 10.3390/ijms19103000 (PMC6213220; doi:10.3390/ijms19103000)
Supplement: Supplementary file 1 [file ijms-19-03000-s001.zip › Supplementary Figures.docx]

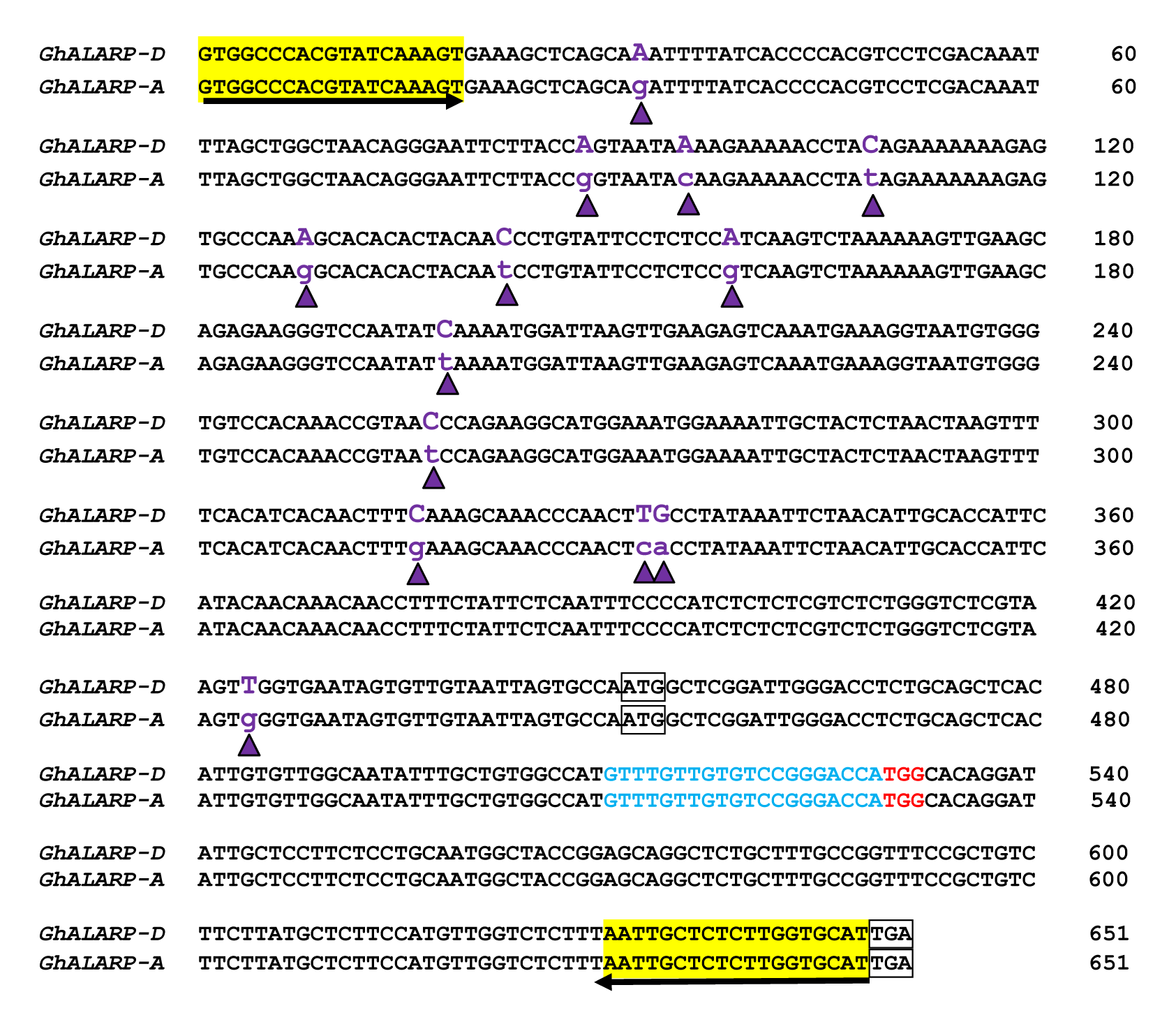


**Supplementary Figure S1.** Identification of *GhALARP*-sgRNA target sites and the primer sites for PCR amplification of DNA in the two different genomes. Blue nucleotides indicate the target sites, red nucleotides indicate the PAM sequence, black arrows and yellow background nucleotides indicate the detecation primer sites, and purple nucleotides represent single nucleotide polymorphisms. Box nucleotides indicate translation initiation codon and termination codon.

**
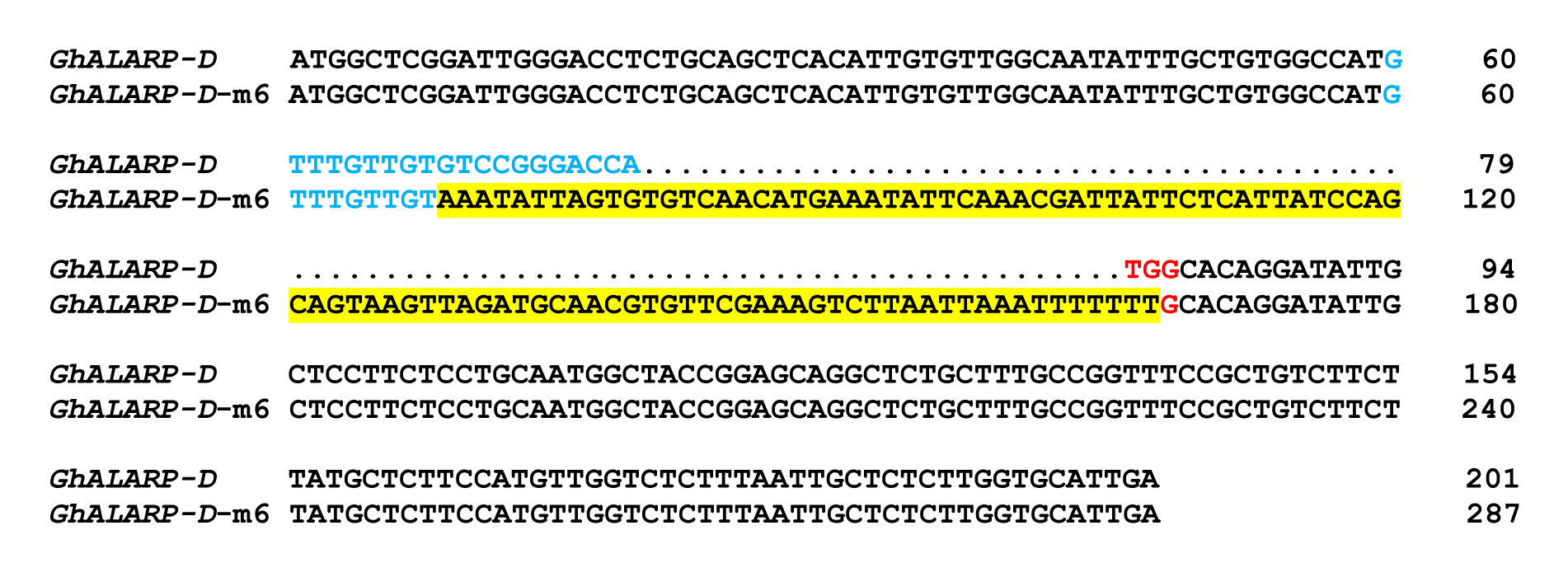
**

**Supplementary Figure S2.** Sequencing analysis of CRISPR/Cas9-induced large fragment insertion mutations (-13/+99; 3×) PCR products at *GhALARP-D* subgenomic sites from the L2 independent transgenic cotton event. Blue nucleotides indicate the target sites, red nucleotides indicate the PAM sequence, and yellow background nucleotides represent the inserted nucleotide sequence.

>D02:-57231491(572 bp)

ATTGCGTCGTTGGATTGGAGATGGTTGTTTGATTCAGTGGGTCATCAATCACTTGATTATTTCCTTCATTCGGTCTATGAGGGTGAGATCACTGTGAAGCCACTGGATGAGGTCTTTGAAGAAGGAGCTCGACGTTGGCAACACTCTATTGTTGCTCAGATCTTTGGAAAGGCCCCGAATTTTAGGTTGACGCAACGGGTTGATGCTATTCTCTAGGGTAACCGAGGTGAGGTTTCTGCTCAATTGGATGGGGATAGTCTTTTCATCTTTCAGTTTTCTTCTGTTAATGCTTATGATTAGGTCCTGGGGAATGGACCCTGGCACATAAAGAACAAACCCTTAATTTTGCGAAAGTAGGAGCCTAGTTTAAAGAATCTTGAGCCTAAGGTATCTAATGTAATTGTTGATGATTTTGTTGTGTCTGGGTCTATGGTTGATGGTTTTAATCTTATGGTTGTAGATCTTGGTTCTATGGCAAGTTTGAATAGTCATGTGGCTACTGCTACTCAAATGGATACATAAGGGTATTTGAGGCCTCACCGAGAGTCTGCTAAAGGGTTAGGGGCTTTGGT

>A11:+20565332(482 bp)

TGTTGTCGTTGAGGATGGGAAACTAGATCCCCTGAATAGGTGAGTCTTTTACAAATATTTTCTGATTTTTTCTTTATTTGTATAGTTTACCCTAAAAGTAGTAACAAGGAGGTTGATGTGGGTTAAAGGATCGACACTACCGACAGGCCTAGCTCTTTTTCCTCATTTGTGCTAGATAGTGCCCATGCTATAATCTCCTTTTATTCTACTGCTTGTGTTTGTTGTTTCAAGGACCAGGGAACGATTAATCCTCACTTTTTCTCTTACGAATTTGTGTGGAAGATTTGTCAATACTGTTGTTGCATTTAGAACATCCAGATGGCTCTCCCTAATGACCATATTCTTTTCTTTAATGCTTGGATGTCTTCTTTTCCCTTCAAAAGATTCAATTTTTTGTGGAAAATGACTTTCTATGCAATTGTGTGGTATGTATGGCTTTGCAAGAATGAAGGTGTGTTTAATGGCAAGGGTGTTGATGTAGC

>D02:+21785633(574 bp)

ACGAAGGAGGCTGTGACATTTGGACAAGATAAGTCATCGAGGATAAAGGAAGCGAGGTGAGTTTCTATACTCTACTTTTGGGTGATCAATGATGTTGTGTTTTTAGTTAAATTAACCCCATGCCTGTCCTGTGTTGTGTCCTAGACCATGGTTG(/T)TGTATAAAAGTGATGAATCTA(/G)TATGCTAC(/T)GGATGCATAACCTCATAAAAGTATGTGAATGTTTGTAAGGATGCTTGATAATTGTCTGGTGGTTACCTCACGATGTTTGAATATTTGATGTATTGCT(/G)TGTTTGTAAATAACACCTTATTTTTGTGTGAAATGCTTGTTCATTATATATGCAAAGTACAGAGGAATATACCTTGACGGGGTATATGGAGATAGGAATAATTGGCACAGTAGAGGAATGGGCAGATTGAAATGGGAATTCAAAGGAGTTAAAC(/T)AACATCGTGGAATGGTTAGCTAATGGCTCGCTAAAATAAAACTATGATGAAAGTTATCGACTAGTTCTTTAGGGCAACATCGTGACGAAGGTTTATAGGTTCCATAGAGCAAGACCGCGACGGCAGTCTACAGGCTCTAT

>D09:+7375255(352 bp)

AAGGAGGCAGTAGTGGTGTCTCCTACTCCTCCTGTTAAAAAAACAAGTCCATTTGCTTTTTCTAGTAACAGGTTTGATATTCTGAATGCTGAGTTACCTGTGGTATTTGATGTAATTATTGATGATTTTGTTGTGTTTGGGACTATGGCTGATGGTTTTGATCTTGTGGCTATAGATCCTGGTTCTGTGGCAGGTTTGAATGATCTTATGGCTACTACTACTTAGATGGATAAAGAAGGGTATTTGAGGCCTACTCGAGAATCTGTTAAGGGTGTAAAGGCTTTAATGAATCTTTAAAGTCTAAAAAGAAAAGGATTGTGAAGAATAAAAAAGTTGTGGCAGGTTCAATTGT

>A10:-6696373(*Gh_A10G0542*) (748 bp)

TCATTGCATGTCCACCTTGTATCAAGTAGTTGGCTTACATGTGAAATATGCACTAGTCTAGTGTTTGATGTATTTCCATCGATCTCAGAGAACATGAGGAATATATAAAAATCATTGAGTCTAGTGTTTGATGTATTTCCATCGATCTCAGAGAACATGAGGAATATATAAAAATCATTGAGTCTAGTGTTTGATGTATTTCCATCGATCTCAGAGAACATGAGGAATATATAAAAATCATTGAGATTGTTAGTCGACATGTATCCTTTTAAAGATGTAAAGTTTATTATGGGGCCTATTTAAGATGTACCAAGAGATTTGGATGAAATCATTCTCTAAGTTGTTTATAGCTTTTCCTTGCTTTCCCTCAAAACACTGCAACAAAGGAACTCTTTCTTCATAATAAACAAATATTAGTGGATTTGACTATACTTGTATTAACATATGGTTGCACATTTGGTAACTATTCTTATTCTCAAATTATGCTGGCCCAAGTGTCCGTTGTTTGACCTGAGTCCTGAAGTATATTGGAAGTATTTGTCATTCCTTTTTGTTGTGTTAGGGATCAGGGGGAAGGGATATGTTTTCCATTATCTTTTGTTTGTACAATTTTAATTAACTGAAGTCCTAATCTACTTAGTGGGTTCACTCGTAATCTTTGTAATTTGTTCCCTCTTCATTAGCCTTGCAACGTAGGACTCTTACCTAGTTAATTTCTGTTCTTTCAGGGTCACAGTGCTGGGCTATT

>D08:+5937364(364 bp)

GCACCCAACTCAATCAACCTGCAATCATTACCTGCTGATTGGATCGGGGTTTAAAAATAAATTTTGTTGGTGCCGACTTTGCAATGGTCGATACTCATATGTATATTTTTGTATACCTTTTGAAAGATACGAGTATTTTTCGGTTAAAAAATATTTTTTATTAAGTGCCAACGTCCACGTGGCTGGCACTATCTATGGAAATTGAAAAAAAAATTGGTAATTTTTTGTGTCGGCATTATGGTGGCCGGCACCCGTGCCAGAAAATAAATTTGGTCCTGTGTTTCTTGTGGCCAGGACCCGGGTAAAAAAAATTAATTTTTTTTGTTGTCGGCCTTCCCCATGTCGGCACCTGAGGATTTGTCAA

>D10:-36547470 (664 bp)

TCTTCTCAACGAACGCACAGGTCGATAGAAGGCAAGTTTTTTTTCTTTTTTATTATTGTTATATTTTTTCTCCTCAAATTTTGATTTTGGTTATTGTTTTTTTCCTTTTCTTTTCGTTTTCATTGGATTTCTCTTCATATAATTTTATTTAAGAACTTGAATCATGTTGCATTTTTCCTAGATCTCATTTCGTTTATTAATTAGATATATTATGATATAATTATTGTAAAAACCAATCATTTATGTAATTGAGTCTTAAAAATTGGTCGGATGGTTCCTCAAAAGATCAAACTGAGCTGAATGTATAAGGATGATAAAAACAAATACCTTTTGGGTTTGAAGGTGCAAAAAGACAATTGTTGAATACTCTTTTCATGAAAGTTCAAATCTTATTCGTCTTACATTACATGATTACTTCTCCATTTTATGGTAAAGCTACATAATGGAACAATGAAATTGTTGGCCTTGTTTTTATAGATGTAATAATCTAAGTACAACTTGATTAGAGTTGGTGCTTGGAAATTGTTCTGCTTGGAATTTTTGTTGTCGTGTGGTACTTAGAAAACATTTTGTTGTGTCCAGGAAAATGGTGAAAATGTGCACTCATGTGTATACAATTTCTTTAGGGGTTGATTCTC(/T)C(/T)ACCATTGTCTGGTAACTGAGGGTCG

>scaffold2307_A09:-61148 (*Gh_A09G2459*) (940 bp)

TAAGGGGAGTTTTTGTTATGGGTACTTGAATTGACAATTATTTTTGTAAGAGGTCAGTATCCATGTATTATTTTGTGTGTAGGATTTAATACTATCCTATATTCCATATCTTTACTTTTGCATGTTGGTTCTTTGATGCAAATCTGATCTTCAATTGATTAAATTGTATGAACTAAATCTGGGTGTGAATATCATATAAACTTTGCTTTTTTGTCTGATTCTAAGACCTGCTAAAATCCTTTATTTACCTAATCTGCAATTTGAAAGGAGCTTTGGTTCTTGGGTAAACTGTACTTATTGTTTGCTCTTATTCTCACCCTCCATCCATGAATTTCTATATTTACATGCTACTTCACAATTAATATAATTTTATAATTTGTTGGTAAATATTGAAAGAGGACCCAAGGTACATACTAGGATTTTCTTTTCTTTTCTTTTTATAGATCTAATAATCTAAGTACAACTTGATTAGAGTTGGTGCTTGGAAATTGTTCTGCTTGGAATTTTTGTTGTCGTGTAGGTACTTAGAAAACATTTTGTTGTGTCCAGGAAAATGGTGAAAATGTGCACTCATGTGTAGACAATTTCTTTAGGGGTTGATTCTTTACCATTGTCTGGTAACTGAGGGTCGACAAAATCGAATAATGAACTATTTTCTTAATGCTTTGGCTTAGCCTTTTAGCTTCTGCACAACTGTGGCATATCTTAGCCTTTCTCTATAAGATTTGTTCAAATTTCTTTTGCCTTTTGTTTGAAAGTATTTATGCTTATATTATTTATCTAAATATTATCTAACCAATTTTATGGCAATTCTGTTTTGGATAATTAAGCACCACTT(/C)TGAGTGCATTTAAAACATATTTCTTGTTCCAACCCTTTAATTCCTCCATCTACTCAAGCAATTGAAATAATAACTTATGAGCAGCACTATCAGCTATTATCA

>D08:-59028209(317 bp)

AGGTAGCGTTGGTGTCAGAGGCGGCAGCGTACCACTTGCCCTGCTGATGTGATGATGGCGGCGGCAAGGCTAAGCTAGGGTTTCAGAAAATTGAAACCCTAGTTTGACTAATCTTCTTGGGTCAGATTTGGGGTTTGGGCTAGATATTGGGCTTGGGGTATTGGATTAGGCTAGTTGGGTTTAATTGTTTTTGTTTTGTTTGTTGGGCCCGGGAAAATGGGCTTGTACAGTTATCACCCTAGCTGAGTGGTTAAAGGCTCTAATGATTACTCATAGTGCGTAGGCCTCAATTGAAATAGCAGCAACTCTAGAAGGCA

>D08:-59042750(367 bp)

TGGGCTTGGGGTATTGGATTAGGCTAGTTGGGTTTAATTGTTTTTGTTTTGTTTGTTGGGCCCGGGAAAATGGGCTTGTACAGCTGCCCCTATTTGCTCGTTGTCGTGTAACGAGAACAGAGCAAAGACTAAGAAAGACCAATTTGCCCGGTCTCGCCGAGTCTTAACTTCTCTTGACGCTTCTCTTCAAGTAACTTCACTCCAGTCCACTGTGTCTTGTTGCTTTGATCCATTCCACTGCACTTCAAGGAGATCTGATTTGTATCTTCAATCTTCTTCGCAGCAATTTCAGGGGGACGAGATTCGTGATTTAAATTTGCTCCACTGCTATGTCAGGGAGATAGGATCGGTTTCTTTGGTCTGCTCC

>D04:+37760986 (563 bp)

GCTTAGCTGCCAGGTCAAAAGAGGCTAGCGTTAGGACAAGCTAAGCTATTAAGGACAAAGGAAGCAAGGTGAGTCTCTACGACTGAATTTTCGGTTATTGTTAATGTTGTGTTGTTTGTTTAAACCACATTCGTGACTTTTTTTTGTTGTGTCCTAAACCATGGTCATGTATGAATATGAAGGTAAGTAAACT(/C)GTGCATGCATATGTTTGCAAGTATGTTCTTTAAGTGTTATATGATTGCGAAGTGATGAATAGATGTATTCTAAAACATATATGATAGCGACTAGCTTGACGTATTGCTTGAAATATACGAGTATA(/G)GTGCATACGGAGTACGGAGGTATTTACCTTGACGGGATATATGAAGTTAGGGATATTGGCAGAATGGACGATTGGGTGGATTGTTGAAGGTATACAGGGGAGTATGGTTATGCCGTGGATTGACAATGTCAGGCTTGCTAGAGTGAAACTGTGACAATAGTTATCAACTGGTCCTTCAAGGTGGCACCCTGATAATGATTTATAGGTTCCATAGAGCAACACCGTGAGGGTGGTTTGTAGGCTCT

>scaffold27980:+36 (476 bp)

AGGTACTTAGAAAACATTTTGTTGTGTCCAGGAAAATGGTGAAAATGTGCACTCATGTGTAGACAATTTCTTTAGGGGTTGATTCTTTACCATTGTCTGGTAACTGAGGGTCGACAAAATCGAATAATGAACTATTTTCTTAATGCTTTGGCTTAGTCTTTTAGCTTCTGCACAACTGTGGCATATCTTAGCCTTTCTCTATAAGATTGGTTCAAATTTCTTTTGCCTTTTGTTTGAAAGTATTTATGCTTATATTATTTATCTAAATATTATCTAACCAATTTTATGGCAATTCTGTTTTGGATAATTAAGCACCACTC(/T)TGAGTGCATTTAAAACATATTTCTTGTTCCAACCCTTTAATTCCTCAATCTACTCAAGCAATTGAAATAATAACTTATGAGCAGCACTATCAGCTATTATCAATTAAGTTAATATAATATATTTACTAAAAATAGGTTGGGTTATTAACGTGGAAC

>A10:+36113181 (340 bp)

CTTTTGGGTTTGAAGGTGCAAAAAGACAATTGTTGAATACTCTTTTAACATGAAAGTTCAAATCTTATTCGTCCTACATGACATAATTACTTCTCCATTTTCTGGTAAAGCTAAAAAATGGAACAATGAAATTGTTGGCTTTGTTTTTATAGATGTAATAATCTAAGTACAACTTGATTAGAGTTGGTGCTTGGAAATTGTTCTGCTTGGAATTTTTGTTGTCGTGTAGGTACTTAGAAAACATTTTGTTGTGTCCAGGAAAATGGTGAAAATGTGCACTCATGTGTATACAATTTCTTTAGGGGTTGATTCTC(/T)TACCATTGTCTGGTAACTGAGGGTCG

**>**scaffold2106_A08:-632710 (445 bp)

TTGCAGGCTCACTCAACAAGTACCAC(/T)AAACTCTTTTATCTCAAGTATGCCAACCAACAGTCTGATATATTCATTCAACCCATCTTCAAATCTTTTACACATTATGGCTTCAGTAGAAACACACTCTTGGGCATACCGACTGAGTCTTACGAATTTTCGCTCTACTCCGTCATTGTCATCCTACCCTATTTCAGCTNACTGAGTCTTACGAATTTTCGCTCTACTCCGTCATTGTCATCCTACCCTATTTCAGCTCTAGAAATTCTTTACGCTTCTGGTCGATAAATCTCTGAATGATATATTTCTTACGGAACTCAGTTTGAAAGAATTCCCAGGTCACCTGCTCTCTCGGAACTACTGATACTAGGGTATTCCACTAGTGGTATGTTGTGTCTCGGAGCAAGGATATAACACATTTTAAACATTCATCTAGAGTACAGGATAGCTCATCAAACAACGTATGGTATTATCAAGCCAGAACTCCGTCCGCTCAGTGTCATCATCA

**>**A11:-87746479 (*Gh_A11G2627*) (759 bp)

ACGTAAGACCAATCTGCATGTTCAAGAATAGGTGGTTCTTTTTTTTGGAGTACAGGTCGTTTATGGAGCATTTCTGGTGGTTCTTTGTGTCCAGAACCAGGGGACTGTTTTCCTTGGAGTAGACGTTGTTTATCTAGAATTTCTGATGGCTCTTCATGTCCAGCAACAGGGGGCTCCTTTTCTTGGAGTAGACGTGGTTTATCTAGCATTTCTGACAGTTCTTCATGTCCAGCAACAGTGGACTCATTTTCTCGGAGTAAACGGATATTATCTAGCACTTTCAACGGCTCTTCATGTCCAAAAATAGGGTGCTCAACTTCTTGGAGTCGACGTCGTTTATCTAGCATTTCCGACGCCTCTTCATGTCCAGCAACATGGAGCCCATTTTCTTGGAGTTGACGCCGTTTATCTAGCATTTCCGAGGGCTCTTCATGTCCAGCAACATGAAGCTCATTTTCTAGCATTTTCGAGGGCTCTTGATGTCTAACAACATGGAGCTCATTTTTTTGGAGTCGACGTCGTTTATCTAGCATTTCAAAGAGTTCTCTGTGTCTAGCAACATGTAGCTCATTTTCTTGGAGTCGATGTCTTTTATATAGCATTTTCGAGGGCTCTTCATGTCCGTCAACTTGGAGCTCATTTTCTTGGAGACGATGTCTTTTATCTAGCATTTTTGAGGGCTCTTCATGTCCATCAACATTTTCCTTGAGTCGACGTCTTTTATCTAGCATTTCAGAGGGCTCTTCGTGTCCATTGATA

**Supplementary Figure S3.** DNA sequences of the 15 detected potential off-target sites. Blue nucleotides indicate the potential off-target sites, red nucleotides indicate the PAM sequence, green nucleotides represent potential single nucleotide polymorphisms, black underlined and yellow background nucleotides indicate the detecation primer sites, and purple nucleotides represent the potential artifact of genome assembly.
