# Supplementary material for: Highly Efficient Targeted Gene Editing in Upland Cotton Using the CRISPR/Cas9 System
Source: Int J Mol Sci. 2018 Oct 1;19(10):3000. doi: 10.3390/ijms19103000 (PMC6213220; doi:10.3390/ijms19103000)
Supplement: Supplementary file 1 [file ijms-19-03000-s001.zip › Supplementary Tables.docx]

**Supplementary Table** S**1.** Analysis of the 19 potential off-target sites.

| Sequence of the putative off-target site | Putative  off-target  locus | Number of  matching bases  (include PAM) | Gene name | Region | Tested? |
| --- | --- | --- | --- | --- | --- |
| GTTTGTTGTGTCCGGGACCATGG | scaffold222153:-146 | 23 | *Gh_Sca222153G01* | CDS | NO |
| TTTTGTTGTGTCTGGGTCTATGG | D02:-57231491 | 19 |  | Intergenic | YES |
| GTTTGTTGTTTCAAGGACCAGGG | A11:+20565332 | 19 |  | Intergenic | YES |
| CTGTGTTGTGTCCTAGACCATGG | D02:+21785633 | 19 |  | Intergenic | YES |
| TTTTGTTGTGTTTGGGACTATGG | D09:+7375255 | 19 |  | Intergenic | YES |
| TTTTGTTGTGTTAGGGATCAGGG | A10:-6696373 | 18 | *Gh_A10G0542* | CDS | YES |
| GTTTCTTGTGGCCAGGACCCGGG | D08:+5937364 | 18 |  | Intergenic | YES |
| TTTTGTTGTGTCCAGGAAAATGG | D10:-36547470 | 19 |  | Intergenic | YES |
| TTTTGTTGTGTCCAGGAAAATGG | scaffold187744:-240 | 19 |  | Intergenic | NO |
| TTTTGTTGTGTCCAGGAAAATGG | scaffold2307_A09:-61148 | 19 | *Gh_A09G2459* | CDS | YES |
| TTTTGTTGTGTCCAGGAAAATGG | scaffold27980:+36 | 19 |  | Intergenic | YES |
| TTTTGTTGTGTCCAGGAAAATGG | A10:+36113181 | 19 |  | Intergenic | YES |
| TTTTGTTGTGTCCTAAACCATGG | D04:+37760986 | 19 |  | Intergenic | YES |
| GTTTGTTGGGCCCGGGAAAATGG | scaffold208662:+246 | 19 |  | Intergenic | NO |
| GTTTGTTGGGCCCGGGAAAATGG | D08:-59028209 | 19 |  | Intergenic | YES |
| GTTTGTTGGGCCCGGGAAAATGG | D08:-59033434 | 19 |  | Intergenic | NO |
| GTTTGTTGGGCCCGGGAAAATGG | D08:-59042750 | 19 |  | Intergenic | YES |
| GTATGTTGTGTCTCGGAGCAAGG | scaffold2106_A08:-632710 | 18 |  | Intergenic | YES |
| GTTCTTTGTGTCCAGAACCAGGG | A11:-87746479 | 18 | *Gh_A11G2627* | CDS | YES |

Mismatching bases are shown in red.

**Supplementary Table** S**2.** The primers used in amplification of the potential off-target sites in this study.

| Primer name | Sequence(5'-3') |
| --- | --- |
| D02:-57231491-F | ATTGCGTCGTTGGATTGGAG |
| D02:-57231491-R | ACCAAAGCCCCTAACCCTTT |
| A11:+20565332-F | TGTTGTCGTTGAGGATGGGA |
| A11:+20565332-R | GCTACATCAACACCCTTGCC |
| D02:+21785633-F | ACGAAGGAGGCTGTGACATT |
| D02:+21785633-R | ATAGAGCCTGTAGACTGCCG |
| D09:+7375255-F | AAGGAGGCAGTAGTGGTGTC |
| D09:+7375255-R | ACAATTGAACCTGCCACAACT |
| A10:-6696373(*Gh_A10G0542*)-F | TCATTGCATGTCCACCTTGT |
| A10:-6696373(*Gh_A10G0542*)-R | AATAGCCCAGCACTGTGAC |
| D08:+5937364-F | GCACCCAACTCAATCAACCT |
| D08:+5937364-R | TTGACAAATCCTCAGGTGCC |
| D10:-36547470-F | TCTTCTCAACGAACGCACAG |
| D10:-36547470-R | CGACCCTCAGTTACCAGACA |
| scaffold2307_A09:-61148 (*Gh_A09G2459*) -F | TAAGGGGAGTTTTTGTTATGGGT |
| scaffold2307_A09:-61148 (*Gh_A09G2459*) -R | TGATAATAGCTGATAGTGCTGCTCA |
| scaffold27980:+36-F | AGGTACTTAGAAAACATTTTGTTGTG |
| scaffold27980:+36-R | GTTCCACGTTAATAACCCAACCT |
| A10:+36113181-F | CTTTTGGGTTTGAAGGTGCA |
| A10:+36113181-R | CGACCCTCAGTTACCAGACA |
| D04:+37760986-F | GCTTAGCTGCCAGGTCAAAA |
| D04:+37760986-R | AGAGCCTACAAACCACCCTC |
| D08:-59028209-F | AGGTAGCGTTGGTGTCAGAG |
| D08:-59028209-R | TGCCTTCTAGAGTTGCTGCT |
| D08:-59042750-F | TGGGCTTGGGGTATTGGATT |
| D08:-59042750-R | GGAGCAGACCAAAGAAACCG |
| scaffold2106_A08:-632710-F | TTGCAGGCTCACTCAACAAG |
| scaffold2106_A08:-632710-R | TGATGATGACACTGAGCGGA |
| A11:-87746479 (*Gh_A11G2627*)-F | ACGTAAGACCAATCTGCATGTTCA |
| A11:-87746479 (*Gh_A11G2627*)-R | TATCAATGGACACGAAGAGCCCT |
